# Portable sequencer in the fight against infectious disease

**DOI:** 10.1038/s10038-019-0675-4

**Published:** 2019-10-03

**Authors:** Arthur Elia Mongan, Josef Sem Berth Tuda, Lucky Ronald Runtuwene

**Affiliations:** 1 Faculty of Medicine, Sam Ratulangi University, Manado, Indonesia; 2 Graduate School of Frontier Sciences, The University of Tokyo, Kashiwa, Japan

**Keywords:** Genetics research, Bacterial infection

## Abstract

Infectious disease is still a major threat in the world today. Five decades ago, it was considered soon to be eradicated, but the adaptation of pathogens to environmental pressure, such as antimicrobials, encouraged the emergence and reemergence of infectious disease. The fight with infectious disease starts with prevention, diagnosis, and treatment. Diagnosis can be upheld by observing the cause of disease under the microscope or detecting the presence of nucleic acid and proteins of the pathogens. The molecular techniques span from classical polymerase chain reaction (PCR) to sequencing the nucleic acid composition. Here, we are reviewing the works have been undertaken to utilize a portable sequencer, MinION, in various aspects of infectious disease management.

Infectious disease has always been intertwined with human history. Poliomyelitis was already documented in Egyptian papyrus. Leprosy, plague, cholera, yellow fever, influenza epidemics, or pandemics were the norm [1]. As the civilization went forward, the development of microscope allowed the visualization of microorganisms for the first time, changing the paradigm of infectious disease [2]. Soon, the discovery of pathogenic agents displaced the preexisting theories of the cause of infectious disease. Vaccines were developed to prevent contracting or spreading the disease and methods of food sterilization were deemed valuable for disease control.

The fight against infectious agents was so efficient that in the 1960s, control and prevention measures had decreased the incidence of many infectious diseases [1]. Smallpox and rinderpest were controlled from the world. Nevertheless, emerging and reemerging infections are becoming significant worldwide problems. Pathogen adaptation seems to hold the key for the emergence or reemergence of infectious disease. The acquisition of drug resistance, for example malaria parasites to chloroquine or the mosquito vector to insecticide, is one of the factors that prevent the eradication of malaria worldwide in 1955–1978 [3]. Other example is the human immunodeficiency virus emergence in the past century due to simian immunodeficiency virus coincidental transmission to humans [4–6] and the 2009 H1N1 pandemic influenza virus that emerged from pigs, is a legacy of influenza A virus causing pandemic in 1918 [7].

The definitive diagnosis of many infectious diseases still relies on direct observation of the causing pathogens. However, at many times this is difficult to achieve, especially for viral diseases because of the size of virus particles. For example, the microscopic examination of acid-fast bacilli remains the main tool for tuberculosis detection, yet the technique sensitivity varies on smearing, staining, and slide reading [8]. Parasites such as microfilaria depends on the sampling time for positive observation [9]. Therefore, molecular techniques, such as PCR or enzyme-linked immunosorbent assay have been developed to assist in a more reliable diagnosis. They are designed to detect the presence of infecting pathogen molecularly. Because it directly measures the nucleic acids or proteins of infecting pathogens, the risk of missing the organism in direct observation may be avoided.

Traditionally, laboratory test for drug resistance is a long and laborious process. First, culturable microorganisms must be cultured on various growth mediums. The time needed for colonies to form also varies greatly. Then the colonies must be subjected to different type of antimicrobials until the susceptibility or resistance can be distinguished. In certain cases, drug resistance analysis is very critical to patients’ prognosis, therefore traditional process is impractical. Here is the niche for genetic analysis to expedite the drug resistance tests.

There are many genetic analyses designed to confirm diagnosis or to test for microbial resistance. PCR can be used to detect the presence of pathogen DNA. Using multiplex primers targeting DNA of many organisms, an investigator can detect the presence of single or multiple infecting agents [10]. Further investigation using primers targeting the presence of known mobile genetic elements can decipher the antibiotics resistance that may be present in the clinical sample [11]. PCR can also be used to check for single nucleotide polymorphisms conferring drug resistance. By creating primers matched the substituted nucleotide, the susceptibility or resistance can be determined by the absence or presence of certain bands [12]. Combination with real-time PCR using probes specific to mutations will amplify mutated gene and can be observed in real time [13].

Sequencing of PCR amplicons or whole pathogen genomic DNA can be applied to comprehensively screen for infecting agents and their drug resistance phenotype, if exists, at the same time. Sequencing technique has been around since 1975 by the works of Frederick Sanger, Allan Maxam, and Walter Gilbert [14]. Since then, the technology has been modified and refined with the automation of the original technique, the inception of next-generation sequencing, and the advent of long-read sequencing. With each iteration, the data throughput is increased, cost is reduced, and physical form of the sequencer is reduced. Sequencing ensures detection of DNA composition unique to an organism, the presence of antimicrobials-destroying genes, or mutation that can change the function or conformation of proteins related to drug resistance. Further application of sequencing using the next-generation platforms encompasses epigenetic profiling as well [15].

Next-generation sequencing incorporates many technologies, but mainly they include the incorporation of terminating nucleotides to a DNA polymerization process [14]. These termination nucleotides can be paired with excitable compounds that is excited by laser beam; and depending on the compound, the excitation process will emit different wavelengths which can be identified as different nucleotide. A different kind of DNA sequencer uses nanopore protein to recognize the nucleotide in question. Here, the disturbance in the basal electrical current inside the protein caused by certain nucleotide strands will be recognized as specific patterns and converted into sequences of DNA by specific algorithm [16].

The deviation from common sequencing principles allows the new sequencing platform to be miniaturized (Fig. 1). This opens new powerful aspects of sequencing that could never been achieved before. The portable sequencer, MinION, can be transported to countries or locations where performing sequencing is difficult or transferring samples to other countries or locations is forbidden. For example, MinION has been taken to a remote rainforest of Tanzania [17], Ecuador [18], the Canadian high Arctic [19], and even the International Space Station [20]. MinION helped the surveillance and sequencing of Zika virus in the 2016 Brazil outbreak [21]. During the project, it was found that most of the genome were fragmentary. Low titer was correlated to sequencing less than 50% coverage, so ‘tiling amplification’ was used to amplify the whole genome of Zika virus. This technique also was employed in the Ebola virus (EBOV) outbreak in Guinea to get a high nucleic acid concentration for sequencing [22].

**Fig. 1:**
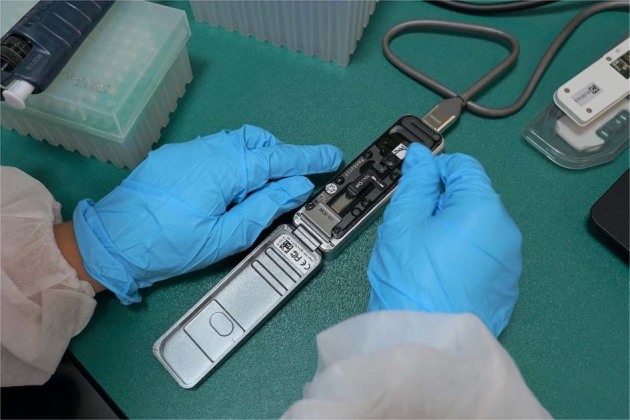
The portable sequencer, MinION, is being handled prior to sequencing

The portability of MinION comes at the expense of sequencing accuracy. Despite recent improvements to the chemistry and computational tools, the sequencing errors are still between 5 and 15% [23]. Consensus calling using bioinformatics tools can improve accuracy to more than 97% [24, 25]. When consensus calling was applied to simultaneously screen for multiple antimalarials resistance, it was clear that north Indonesia, north Vietnam, and southeast Thailand had different mutation patterns in *K13* gene [26], the gene related to artemisinin resistance, that were in concordance with a major paper describing the artemisinin-resistance related mutations around the Mekong region [27]. Nevertheless, the mutations conferring resistance to chloroquine were similar in these three regions. This method applies the amplification of multiple genes with PCR. The sequencing depth is high so consensus sequence can be generated with high confidence. Targeted sequencing is cost efficient by performing multiplex sequencing. Although the running cost of other sequencing platform is cheaper than MinION (for example, $67.82 for MiSeq compared with $71.56 for MinION per sample [28]), MinION has significantly lower cost to set up.

Targeted sequencing with MinION is powerful and fast to detect pathogens in clinical samples. Bacterial composition from empyema patient’s pleural effusion could be understood within 2 hours after obtaining the DNA sample [29]. Here, the amplicons of *16*s *rRNA* gene were sequenced to identify the bacteria. Due to the capability of long-read sequencing, the gene can be sequenced in entirety, increasing the resolution to detect the infecting bacteria species. Bioinformatics tools can be used to increase the accuracy of the amplicons and determine the bacteria composition up to species level [30]. The employment of targeted sequencing using 285 and 256 primer pairs enabled the recognition of the causative agent of viral hemorrhagic fever within 10 min of sequencing, and a definitive diagnosis can be procured in less than 3.5 h [31].

Other techniques can be used to amplify the gene of interest instead of PCR (see Table 1 for an outline of methods employed with MinION). Loop-amplified isothermal amplification (LAMP) is method to amplify a gene segment flanked by two to three pairs of primers in an isothermal condition [32]. Further, LAMP reagents can be dried to assure their stability upon transportation to the field [33]. Applying the LAMP technique as the amplification method, one group of researchers performed a genomic epidemiology study of dengue virus (DENV) in Indonesia and Vietnam [34]. Up to 141 and 80 DENV-positive samples were amplified isothermally and sequenced with MinION. The successful detection rate was 79%. Serotype could also be determined despite the 74–80% identity. The LAMP–MiNION technique can be applied to other pathogens as well, such as malaria [35, 36] and chikungunya [37].

**Table 1:** An outline of MinION sequencing methods and the cited references

MinION sequencing method	Techniques applied	Reference
Targeted sequencing	Amplified with PCR	[21, 22, 26, 28–31]
	Amplified with LAMP	[34–37]
Whole-genome sequencing	Metagenomic sequencing	[38–41, 45–49, 51–53, 59]
	Isolate sequencing	[56–58]
Advanced techniques	RNA/cDNA sequencing	[61–63]
	Epigenetic sequencing	[66, 70, 71]

With the increase of MinION sequencing power, whole-genome sequencing of pathogens [38–40] and human [41] using MinION has become common practice in the field or clinical settings. Metagenomic sequencing, previously limited to high-throughput short-read sequencers, has gotten more applications with MinION [42]. Although the ideal approach is to make no presumptions about the infecting pathogens and sequence all the DNA materials contained in the environmental or clinical specimens, there are many technical difficulties, such as the low titer of the infecting agents [21, 34] and large host DNA background [43]. Sequencing the cultured isolates might overcome the problems. However, because only 1–2% of bacteria can be cultured [44], unculturable bacteria might be overlooked, so are the cases with virus, fungi, or parasite infections.

For metagenomic sequencing, there are several methods can be employed to overcome the difficulties. Host DNA depletion with saponin [45], negative CpG selection [46], or physical disruption of bacterial wall [47, 48] may be used to remove or reduce the background DNA and to increase sensitivity. Filtration, nuclease digestion, and random amplification allow reliable recovery of viral genomes [49]. Whole genome amplification by multiple displacement amplification (MDA) can also be performed for low DNA concentration [50]. This technique uses the high-fidelity phi29 polymerase combined with random hexamer primers to amplify DNA in isothermal reaction. Specific MDA protocols for amplifying Plasmodium DNA from whole blood [51] and Mycobacteria DNA from sputum [52] have been published.

Metagenomic sequencing with MinION has been used in many clinical studies. Chikungunya virus (CHIKV), EBOV, and hepatitis C virus can be detected from clinical samples after amplification of viral genomes [38]. In a similar experiment, a coinfection of DENV and CHIKV was found from one clinical specimen [39]. Pathogens causing lower tract infections can be identified by sequencing bronchial lavage with or without host depletion [45, 53]. This allowed the finding of bacteria composition and drug resistance phenotype in lower respiratory infection, which led to early antibacterial therapy within 6 h [45].

Other advantage of MinION is the ability to physically sequence long reads. Using ultra-long-read sequencing protocol, a read >2 megabases is obtainable with special protocol [54]. This is particularly important for de novo sequencing of clinical samples, which can have a very elaborate gene structures harboring drug resistant genes with high copy number. For example, plasmid-mediated extended spectrum beta-lactamase *Escherichia coli* strains can have large numbers beta-lactamase genes, leading to high-level resistance [55]. The long-read sequencing could assist the investigation of antimicrobial gene location, copy number, and potential transposon-driven rearrangements of *E. coli* [56, 57] and *Salmonella typhi* [58] isolates that otherwise could not be capture by short reads by assembling the genome de novo. Furthermore, field sequencing using MinION of Lassa virus outbreak in Nigeria molded the management response by the government [59]. By confirming the outbreak was caused by the same strains that transmitted from rodents to humans, the government focused the efforts on rodent control and safe food storage. Since Lassa virus is genetically highly diverse, long-read sequencing is the method of choice.

Nanopore sequencing is not limited to DNA. RNA sequencing, either directly or after cDNA conversion, has been widely performed with MinION. Studies in RNA virus will gain substantial benefit because most of the high-profile human viral diseases that recently emerged are caused by RNA virus [60]; sequencing the virus in their natural state can open a lot of exciting findings. Already, direct sequencing of RNA virus and transcripts of DNA virus reveals the structural variants of coronavirus [61], an mRNA that accumulates late in infection of herpes simplex virus-1 [62], and the discovery of novel RNA molecules and transcript isoforms of varicella zoster virus [63].

Advanced application of MinION in the field of infectious disease is the epigenetic sequencing. Epigenetics is the study of heritable changes in the gene expression that do not involve changes in genomic DNA sequence [64]. In infection, the researches are mainly focused on the changes in host’s DNA methylome, histone marks, and microRNA profiles as a response to pathogen’s invasion. Relatively new, the number of infection-related epigenetic researches are still low compared with cancer epigenomics [65]. MinION has a high potential assisting epigenetic research. Due to its principle of directly sequencing nucleic acid molecules, any modifications in the nucleotides are also recorded in the raw signals. Therefore, untreated nucleic acid sequenced with MinION has enough information to distinguish 5-methylcytosine from cytosine if carefully analyzed [66]. Some software have been developed specifically to read these signals and translate them into methylation marks [66, 67].

These developments open many research possibilities by allowing both genome and epigenome to be analyzed from a single sequencing run [68]. For example, 16s ribosomal RNA (16s rRNA) can be sequenced with RNA sequencing. Theoretically, the method can identify bacteria species and screen for antibiotics resistance simultaneously. By identifying gain or loss of ribosomal RNA base modifications, antibiotic resistance can be inferred [69]. Sequencing of *E. coli* 16s rRNA, as well as its DNA has been shown to be capable of detecting 7-methyl-guanosine, pseudouridine, 5-methylcytosine, and 6-methyladenine modifications [70, 71]. The progression of this field may have clinical applications in the future.

Sequencing as a technique may become the gold standard of diagnosis with the advent of portable sequencing. The technology is still evolving but it has shown development in portability, sequencing accuracy, and ease of operation. In portability, new type of flowcell and equipment can enhance sequencing in the field or in the laboratory. Additional equipment such as Voltrax (an add-on for the flowcell which will automate library preparation) or MinIT (a GPU computer) may eliminate the need to for extraction kit or laptop computer. Many methods have been shown to be successful coupled with MinION. If targeted sequencing is needed, existing methods, such as LAMP can amplify genes of interest in an isothermal reaction, eliminating the need for thermal cycler. MDA can amplify whole genome of pathogens to reach the optimum input DNA concentration. The isothermal nature of the enzymes used in LAMP and MDA is also advantageous for field or clinical settings. The sequencing power is now enough for unbiased metagenomic sequencing. All of these will pave the way for the portable sequencer be used in field or clinical setting to assist in the fight against infectious disease.
